# Identifying potential treatment effect modifiers of the effectiveness of chiropractic care to infants with colic through prespecified secondary analyses of a randomised controlled trial

**DOI:** 10.1186/s12998-021-00373-6

**Published:** 2021-04-19

**Authors:** Lise Vilstrup Holm, Werner Vach, Dorte Ejg Jarbøl, Henrik Wulff Christensen, Jens Søndergaard, Lise Hestbæk

**Affiliations:** 1 Nordic Institute of Chiropractic and Clinical Biomechanics, University of Southern Denmark, Campusvej 55, DK-5230 Odense M, Denmark; 2 Research Unit of General Practice in Odense, Department of Public Health, University of Southern Denmark, J.B. Winsløws vej 9A, DK-5000 Odense C, Denmark; 3 Basel Academy for Quality and Research in Medicine, Steinenring 6, 4051 Basel, Switzerland; 4 Department of Sports Science and Clinical Biomechanics, University of Southern Denmark, Campusvej 55, 5230 Odense C, Denmark

**Keywords:** Infantile colic, Chiropractic, Randomized controlled trial, Manipulative treatment, Excessive crying, Effect modification

## Abstract

**Background:**

A recent trial identified large variation in effect of chiropractic care for infantile colic. Thus, identification of possible effect modifiers could potentially enhance the clinical reasoning to select infants with excessive crying for chiropractic care. Therefore, the aim of this study is to identify potential treatment effect modifiers which might influence the effect of chiropractic care for excessive crying in infancy.

**Methods:**

Design: Prespecified secondary analyses of data from a randomised controlled trial. The analyses are partly confirmative and partly exploratory.

Setting: Four chiropractic clinics in Denmark.

Participants: Infants aged 2–14 weeks with unexplained excessive crying. Of the 200 infants randomised (1:1), 103 were assigned to a chiropractic care group and 97 to a control group.

Intervention: Infants in the intervention group received chiropractic care for 2 weeks, while the control group was not treated.

Main analyses: The outcome was change in daily hours of crying. Fifteen baseline variables and 6 general variables were selected as potential effect modifiers, and indices based on these were constructed. Factor analyses, latent class analyses and prognosis were used to construct other potentially modifying variables. Finally, an attempt at defining a new index aiming at optimal prediction of the treatment effect was made. The predictive value for all resulting variables were examined by considering the difference in mean change in crying time between the two treatment groups, stratified by the values of the candidate variables, i.e. interaction analyses.

**Results:**

None of the predefined items or indices were shown to be useful in identifying colicky infants with potentially larger gain from manual therapy. However, more baseline hours of crying (*p* = 0.029), short duration of symptoms (*p* = 0.061) and young age (*p* = 0.089) were all associated with an increased effect on the outcome of hours of crying.

**Conclusion:**

Musculoskeletal indicators were not shown to be predictive of an increased benefit for colicky infants from chiropractic treatment. However, increased benefit was associated with early treatment and a high level of baseline crying, suggesting that the most severely affected infants have the greatest potential of benefiting from manual therapy. This finding requires validation by future studies.

**Trial registration:**

Clinical Trials NCT02595515, registered 2 November 2015.

**Supplementary Information:**

The online version contains supplementary material available at 10.1186/s12998-021-00373-6.

## Introduction

Infantile colic is a condition characterised by excessive and unexplained crying that occurs in up to 20 % of newborns [1]. Although the condition is usually transitory, it can be very stressful for the family as a whole and has been associated with sequalae such as maternal postpartum depression and increased stress levels years after cessation of the crying [2–4]. No standardised treatment exists for infantile colic, but chiropractic care is common [5, 6], despite a lack of solid evidence of its effectiveness for children. Few randomised controlled trials (RCTs) have been conducted worldwide, and most are of low quality [7–9].

Despite several years of research, the pathophysiology of this excessive crying still remains unclear. It is increasingly acknowledged, however, that children with infantile colic do not form a homogeneous group but represent several subgroups with different aetiologies, implying that no one treatment suits all children [10–12]. Hence, each subgroup may benefit from different interventions. RCTs conducted to evaluate the effect of any treatment on infantile colic have typically estimated the effect at a group level, i.e. including all excessively crying babies regardless of aetiology, signs and symptoms, and to our knowledge, no studies have investigated potential effect modifiers of treatment of infantile colic. Admittedly, these RCTs are typically not designed to investigate modifying effects in subgroups, but still the data may be used for explorative analyses to indicate if a treatment is more effective in certain subgroups than in others [13].

The current study is based on data from a randomised controlled trial evaluating the effect of chiropractic care in Danish infants aged 2–14 weeks with excessive crying. The results are in the publication process, but details of the study design are described in the published study protocol [14]. The primary outcome was change in daily hours of crying during a two-week period from start of treatment. In the primary analysis, we found that the mean reduction in crying was half an hour in favour of the group receiving chiropractic care compared with the control group, but not at a statistically significant level after adjustment for baseline values. From a clinical perspective, the mean difference between the groups was quite small, which might be explained by a variety of factors including a limited effect of chiropractic care in this group of children; an outcome that was not responsive; or a heterogenous group of children with different causes of excessive crying, not all responsive to chiropractic therapy. There were, in fact, large individual differences in the effect on crying time, which emphasises the need to investigate if it is possible to identify certain subgroups of children who benefit more from chiropractic care than others. Chiropractic care is concerned with diagnosis and treatment of mechanical disorders in the musculoskeletal system [15], and in the existing literature, it is hypothesised that one cause of infantile colic could be pain originating from the musculoskeletal system [11, 16, 17]. Included in the protocol for the primary study, we formulated the hypothesis that subgroups of colicky children with musculoskeletal problems potentially would have a larger effect from chiropractic care than others. Identification of such characteristics could potentially enhance the clinical reasoning to select which infants with excessive crying should be referred to chiropractic care and which should not.

Therefore, the aim of the current study was to identify potential treatment effect modifiers, including indicators of musculoskeletal problems and other baseline variables, which may influence the effect of chiropractic care for excessive crying in infancy. The study is partly confirmative with respect to validation of selected items and pre-specified indices based on the available variables, and partly exploratory with respect to making suggestions for new indices.

## Methods

### Setting and participants

We used data from an RCT that evaluated the effect of chiropractic care on infantile colic. The trial was a parallel single blinded RCT that included 185 children aged 2–14 weeks, fulfilling criteria for infantile colic and randomised individually from November 2015 to July 2019 on the Island of Funen, Denmark. Inclusion criteria for participation were infantile colic defined as excessive crying for 3 or more hours per day, at least 3 days a week within the past 2 weeks, but otherwise normal development, including appropriate weight gain, and no prior chiropractic care. Exclusion criteria were known disease or suspicion of disease. The protocol and the main results of the RCT are reported elsewhere [14, 18], thus, only a brief description is included here.

### Recruitment

The project manager visited the families of potential participants in their own home, interviewed the parents and assessed the child for eligibility. If the child met the criteria for participation, and the parents agreed to participate, they were instructed to keep a structured diary of the child’s behaviour (including crying, sleeping, feeding and defecation) [19] during a baseline observation period for at least 3 days. The project manager then evaluated the notes from the baseline observation period, and children fulfilling the criteria for inclusion were randomly assigned to either the intervention or the control group. The parents continued to keep a daily diary throughout the project period [14].

### Intervention

All included children attended a chiropractor clinic four times over a period of 2 weeks. On the first visit, the chiropractor completed a questionnaire based on interviews with the parents to obtain anamnestic information about possible musculoskeletal problems. In both groups, the chiropractor undressed the child and observed if there were any visible asymmetries that could indicate a dysfunction in the musculoskeletal system. For children in the intervention group, a full examination including movement palpation of the joints was carried out, and manual treatment applied individually according to any dysfunction found. Furthermore, if relevant, parents were instructed in specific exercises to improve musculoskeletal function, e.g. use light or sound to motivate the child to turn its head towards the side it was reluctant to turn to. Children in the control group received no active treatment and were only instructed in general exercises, e.g. cycling movements of the legs to stimulate peristalsis. To maintain blinding of the parents, they were not present in the room, while the child was or was not treated. The parents handed in their diary and responses to a final questionnaire regarding the status of the colic to the chiropractic clinic 1 to 4 days after the fourth visit, and the allocation was then revealed [14].

### Outcome

The outcome was defined as change in daily hours of crying, comparing the average of the 3 days before the first chiropractic visit with the average of 1 to 4 days after the fourth chiropractic visit based on the diaries kept by the parents [14].

### Potential treatment effect modifiers

When planning the RCT, we hypothesised that colicky children with a possible musculoskeletal cause would benefit more from the treatment compared with colicky children without that cause. However, it was unclear how to identify this condition in a reliable manner, and hence, no corresponding eligibility criteria were defined. Instead, attempts were made to define variables, potentially indicating a musculoskeletal cause, based on the existing literature [11, 16, 17] and knowledge from an expert panel consisting of two maternal and child health nurses and two chiropractors with experience in the paediatric field.

Overall, the following thirteen binary items (answered by yes/no) were identified:

1. Does the child resist to lying prone? (resist prone)
2. Are there difficulties dressing/undressing the child? (resist dressing)
3. Do crying episodes start suddenly? (sudden crying)
4. Is the child able to sleep deeply and undisturbed? (disturbed sleep)
5. Is the child tense during meals? (tense meals)
6. Does the child favour one side during meals? (fav. Side meals)
7. Does the child favour one side when sleeping? (fav. Side sleep)
8. Does the child have increased tonus in the back musculature? (back extension)
9. Does the child have asymmetric gluteal folds? (asym. Glut.)
10. Does the child have asymmetric hips? (asym. hips)
11. Does the child have asymmetric knees? (asym. knees)
12. Does the child lie in a crooked position/c-curve? (c-curve)
13. Does the child have asymmetric tonus in the back musculature? (asym. Back tonus)

Furthermore, we asked the treating chiropractors about their expectation after talking with the parents, but before examining the child. Two questions were asked:

14. Do you believe that the crying could have a biomechanical cause?
15. Do you believe that the child could benefit from chiropractic treatment?

These 15 items are henceforth referred to as the ‘musculoskeletal items’.

As preparation for the current analyses, another panel of 11 chiropractors with extensive experience in the paediatric field were presented with the 13 original items and were asked to select the variables they believed were most likely to be related to musculoskeletal problems in colicky children. The items 1, 6, 8, 12 and 13 were corroborated by seven or more of these chiropractors, whereas most of the other items were supported by less than four of the 11 chiropractors.

Finally, we selected six additional baseline variables to be included in the analysis of potential modifiers. These variables were 1) the baseline crying time, 2) the time since onset of the excessive crying, 3) the infant’s age 4) the degree of breast feeding (bottle-fed / partial breast feeding / only breast feeding), 5) the educational level of the mother (on a six-point scale) and 6) whether the parents reported to have experienced stressful life events, e.g. death or illness in family or friends, unemployment etc., during pregnancy or after birth.

### Data collection

Information on the 15 musculoskeletal items were collected during the first chiropractic visit. Items 1 to 7 were based on anamnestic information and these were registered by the chiropractor prior to the physical examination, and items 14 and 15 were completed at the end of the history-taking. Items 8 to 13 were included in the standard documentation of the initial examination. Item 4 was the only item actually asking for a sign which indicated no biomechanical dysfunction; hence this item was internally coded inversely, i.e. “no” as 1 and “yes” as 0.

The six additional candidate variables, representing background information, were collected by the primary investigator (LVH) during the first home visit.

For four children, information on none of these items was available. For one child, only three items were available. These five children were excluded from the analyses. The remaining children had a maximum of three missing items. Thus, the sample size for this investigation was 180 children.

### Overall analytic strategy

For the confirmative component of the study, we first defined a series of candidate variables. These also included some variables based on a statistical analysis of the distribution of the variables measured at baseline. All these analyses were blinded with respect to the outcome data. In a second step, the predictive value of each variable was examined by considering the difference in mean change in crying time between the two groups, stratified by the values of the candidate variables and assessing the statistical significance of the association between the difference and the values of the candidate variable, i.e. of the interaction.

For the exploratory component of the study, an attempt was made to construct a new index based on the 15 musculoskeletal items. The value of this index was depicted in the same manner, except that the significance of the relationship could not be estimated.

### Descriptive statistics

The distribution of the 15 musculoskeletal items is described by absolute and relative frequencies. The distribution of additional variables and musculoskeletal candidate variables is depicted by histograms. The association between the musculoskeletal items is depicted by odds ratios, and between the additional variables (and musculoskeletal items) by Pearson correlations.

### Elicitated candidate variables

For single-item construct variables, we used all the musculoskeletal items and the additional baseline variables mentioned above under ‘potential effect modifiers’. In addition, we considered three predefined summary measures:

1. Overall index: Sum of items 1 to 13.
2. Chiropractor index: Sum of items 1, 6, 8, 12 and 13, i.e. the items regarded as most informative by the chiropractor panel.
3. Parental index: Sum of items 1 to 7. This would represent an index which might facilitate the identification of children based on information which could be obtained directly from the parents without involving a chiropractor.

When computing these indices, missing values in single items count as 0, as it is likely that parents and chiropractors are quite confident about the presence of an item but may be less confident about its absence. However, for children with more missing than observed values for the items of an index, the index remained undefined.

### Constructed candidate variables

#### Factor scores

The 15 musculoskeletal items may reflect latent variables partially related to the musculoskeletal cause. A factor analysis may identify these latent variables and allow investigation of their predictive values. The factor scores were based on a factor analysis with varimax rotation. The first factors from the analysis explaining more than 90% of the variation were considered, and corresponding scores were computed using the regression approach. The factor analysis was based on the available case correlation matrix to allow all the children to be included.

#### Phenotypical subgroups

The 15 musculoskeletal items and the additional baseline variables allowed us to divide the population into different phenotypes, and determine if those phenotypes might be related to musculoskeletal causes. Hence, it made sense to investigate the effect within each phenotypic group.

The phenotypical classification was achieved using a latent class analysis involving all single items and the additional six baseline variables except for age, as age is highly correlated with the time since onset of excessive crying. The number of classes was determined by the Akaike Information Criterion, as long as all classes included more than 15 children. In the final model, each child was assigned to the class with maximal posterior probability.

#### Prognostic index

To check, whether the effect of chiropractic care depended on the prognosis of the children, we used the data from the RCT to construct a prognostic index. We made use of a simple linear regression including all musculoskeletal items and the six additional baseline variables. With this number of covariates, we reached the critical limit of 10 observations per estimated parameter, and therefore also applied the lasso method [20] to obtain a stable and parsimonious index. Note that by definition, we had to expect an association between the baseline crying level and the outcome.

### Evaluation of the predictive value

The predictive value of a candidate variable was illustrated by reporting the mean values of the primary outcome in each treatment group within each classification subgroup defined by the candidate variable directly or after a suitable categorisation. Categorisations were aimed at defining four groups of equal size. The predictive value was assessed by reporting the estimated treatment effect at two anchor points and the difference between these two effects (interaction) with a 95% confidence interval and *p*-value. The anchor points were the two groups in the case of a binary candidate variable, and the lower and upper 10th percentile in the case of continuous candidate variables. The estimated treatment difference was based on regression models with the baseline crying level as an additional covariate. In the case of the phenotypical classification, each group built an anchor point and only the p-value of the interaction was reported.

### Explorative component: construction of a new index

We used the technique of Tian et al. [21] to construct a new index with predictive value. The method is based on the simple idea of defining a variable corresponding to the observed outcome in untreated children and to the negative of the observed outcome in treated children (after subtracting the overall mean value). Then the new index was based on trying to predict this variable based on the 15 single original musculoskeletal items. Twice the predicted value was then interpreted as the expected gain in treating a child instead of giving no active treatment, and hence we expressed the new index in these values. For constructing a new index, we used ordinary regression as well as the lasso [20] as suggested by Tian et al. [21]. The latter also implied a variable selection, i.e., the lasso aimed at constructing a parsimonious index with high predictive value by selecting an optimal penalty parameter *λ* penalising the number of items included. The optimal value was determined by cross-validation.

## Results

### Confirmative component

#### Distribution of variables and association among variables

**Fig. 1:**
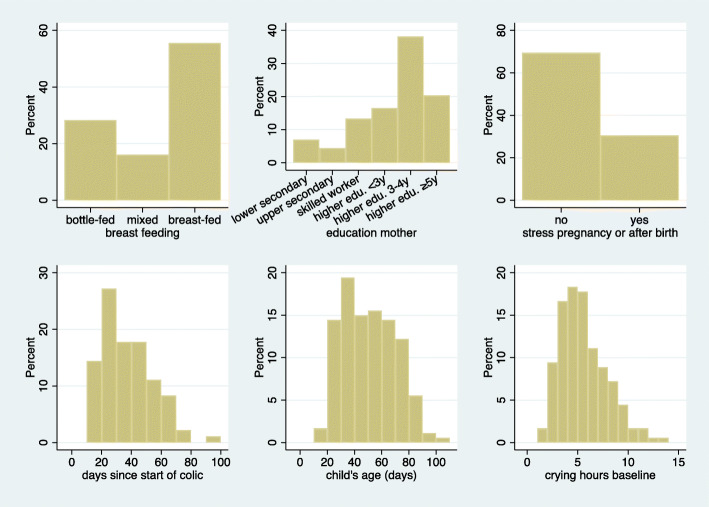
Distribution of six baseline background variables considered as potential effect modifiers

Items 14 and 15 (chiropractors’ expectations) turned out to be highly correlated. For only three children, the answers differed. We therefore decided to merge this into one item, where a positive response indicates a “yes” to both questions. The frequencies of “yes” answers to the 14 dichotomous musculoskeletal items (the 13 preselected items and the additional item about the expectations of the chiropractor) are shown in Table 1. We can observe that asymmetric hips or knees are quite rare conditions. In contrast, sudden onset of crying episodes is quite common. In only 10% of the children, did the chiropractors not suspect a musculoskeletal problem which could be treated. Figure 1 provides information on the distribution of the six additional baseline variables.

**Table 1:** Frequency of positive responses to the 14 musculoskeletal items measured at baseline

Item number		n	Absolute frequency of positive responses (n)	Relative frequency of positive responses (%)
1	resist prone lying	180	80	44
2	resist dressing	180	94	52
3	sudden crying	179	155	87
4	disturbed sleep	178	121	68
5	tense meals	178	96	54
6	favourite side, meals	179	75	42
7	favourite side, sleep	178	105	59
8	back extension	177	61	34
9	asym. Gluteal	178	51	29
10	asym. Hips/knees	179	29	16
12	c-curve	179	136	76
13	asym. Back tonus	178	99	56
14	expectations	177	158	89

The associations among the 14 items showed that expectations of the chiropractor is positively associated with all 13 single items, with highest association with the items 1, 6, 7, 8, 10, 12, and 13, i.e. partially agreeing with the prioritisation made by the chiropractor panel. The other odds ratios are all close to 1.0, with the exception of a high association between asymmetry of hips and of knees, and a moderate association between asymmetric gluteal folds and asymmetric hips and between the two items where a particular side was favoured. Since asymmetry of the hips and knees are both strongly correlated and quite infrequent conditions, we decided to join them to form a single item ‘asymmetry of knees or hips’, thus reducing the number of musculoskeletal items to 13 (Additional Material Table 1). Furthermore, there is a strong association between a favourite side for sleeping and favourite side for feeding. The association between the six additional baseline variables and with the musculoskeletal items was quite low (Additional Material Table 2).

#### Construction of candidate variables

The factor analysis indicated a solution with two factors. However, the first factor only combined items 6 and 7, and the second factor items 12 and 13, i.e. the pairs with highest associations. Hence, we failed to identify broad latent factors associated with several items as intended. This is in line with the low pairwise associations seen in Additional Material Table 1.

The latent class analysis failed to identify any underlying structure. The Akaike Information Criterion decreased continuously with increasing number of classes.

The regression analysis suggested an index with an adjusted R^2^ of 0.08. This was already reached with a model including only the baseline crying level as covariate. Hence, none of the constructed candidate variables seemed to carry any additional prognostic value. Also, the lasso could only identify the baseline level as predictor for the change in crying level, meaning no prognostic index could be constructed.

Hence, all attempts to construct candidate variables were unsuccessful.

#### Evaluation of the predictive value

**Fig. 2:**
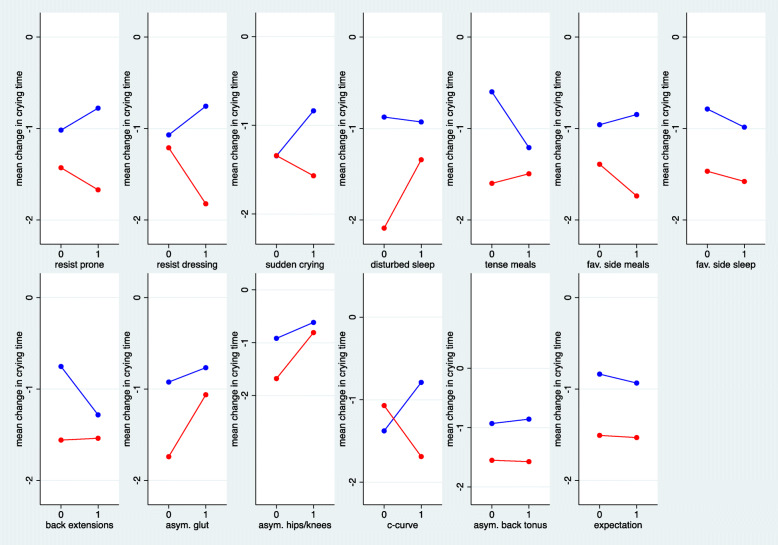
The mean change in crying time stratified by absence or presence of the 13 musculoskeletal items in the treatment groups. (red = chiropractic care; blue = control group)

**Fig. 3:**
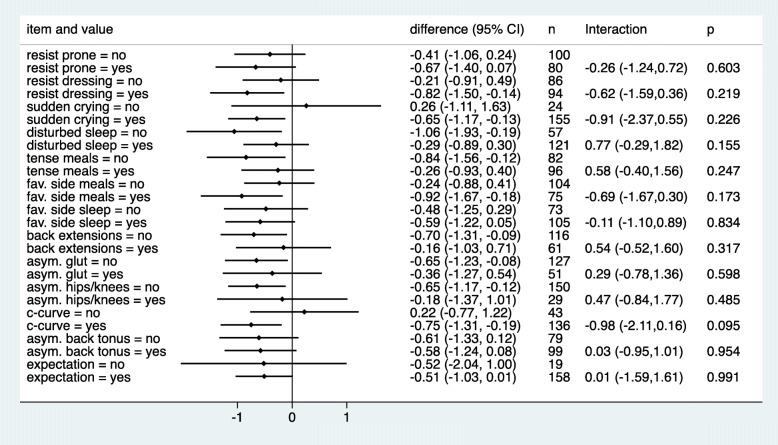
Estimated treatment effects (difference in mean change) in subgroups defined by absence or presence of the 13 single musculoskeletal items. Negative estimates indicate an advantage for chiropractic treatment. Interactions refer to the difference in treatment effects between presence and absence of the item. Negative interactions indicate a more pronounced advantage of chiropractic care in the case of presence of the item

Figures 2 and 3 depict the predictive value of the 13 single musculoskeletal items. In Fig. 2, we can observe that for only eight of the 13 items, the outcome improved under chiropractic care when the item was present. In particular, for the two items about asymmetry, the outcome was worse when asymmetry was present, as well as for children with poor sleep quality. However, this tendency was also observed in the control group for the items about asymmetry. Similarly, in Fig. 3, we observed a negative interaction for six items and a positive interaction for seven items. None of the interactions reached statistical significance. The most distinct interactions could be observed with respect to c-curve and sudden onset of crying, both with no difference between the treatment groups in the case of absence but an advantage for manual therapy in the case of presence. The most distinct interaction in an unexpected direction was observed for sleep disturbance with a bigger advantage for manual therapy in the case of absence.

**Fig. 4:**
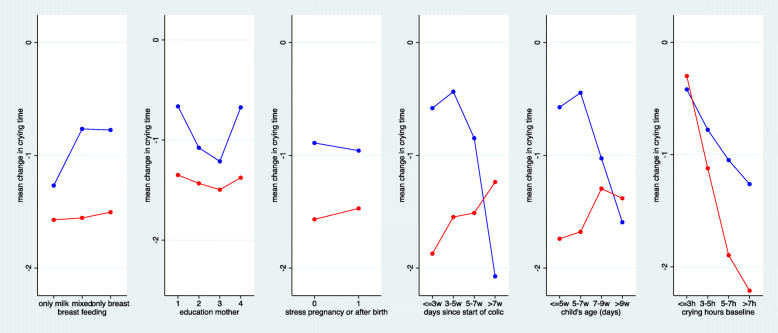
The mean change in crying time stratified by the six preselected additional baseline variables. Duration, age and crying hours at baseline were categorised to obtain four groups of roughly equal size. (red = chiropractic care; blue = control group)

**Fig. 5:**
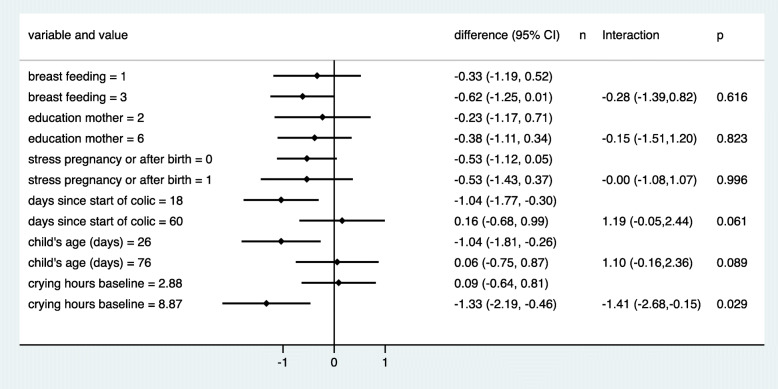
Estimated treatment effects (difference in mean change) at two selected values of each of the six preselected baseline variables. The estimates are based on a model that assumed a linear change in the treatment effect in dependence on the values. Negative estimates indicate an advantage for chiropractic treatment. Interactions refer to the difference in treatment effects between the two selected values. Negative interactions indicate a more distinct advantage of chiropractic care in case of large values. The selected values refer to the 10th and 90th percentile of each variable

Figures 4 and 5 depict the predictive value of the six preselected baseline variables. We observed no influence of breast feeding, education of the mother or stress on the outcome in the chiropractic care group. An early start of treatment and/or young age seemed to favour the outcome under chiropractic care, whereas the outcome was best when starting late and/or at an older age in the control group, probably reflecting that some children were already close to the natural end of the colic period. We observed no treatment difference in children with a late start of treatment, but an advantage of about 1 h with chiropractic care in children starting within the first 3 weeks or earlier. These interactions were not statistically significant (*p* = 0.089/0.061). With respect to crying hours at baseline, we expected a better outcome with increasing hours, as the room for improvement increased and the outcome was defined as the difference from baseline. This trend was indeed very distinct in both treatment arms, but the relationship was more pronounced under chiropractic care. When children cried 3 h or less at baseline, we observed a decrease of less than half an hour in both treatment groups, but when the crying time at baseline was approximately 9 h, we observed an advantage of almost 80 min in reducing the crying time when using chiropractic care. This interaction was significant (*p* = 0.029).

**Fig. 6:**
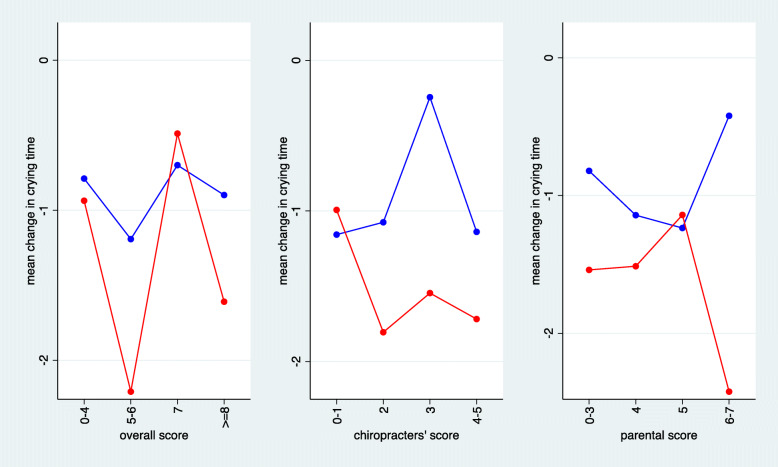
The mean change in crying time stratified by score values for the three predefined scores. The scores were categorised to reach roughly four groups of equal size (red = chiropractic care; blue = control group)

**Fig. 7:**
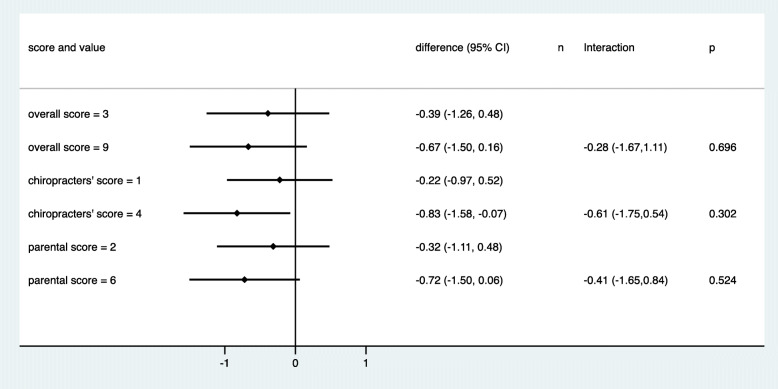
Estimated treatment effects (difference in mean change) at two selected thresholds of the three predefined scores. The estimates are based on a model that assumed a linear change in the treatment effect in dependence on the score values. Negative estimates indicate an advantage for chiropractic treatment. Interactions refer to the difference in treatment effects between the two selected values. Negative interactions indicate a more pronounced advantage of chiropractic care in the case of larger score values. The selected values refer to the 10th and 90th percentile of each score

Figures 6 and 7 depict the predictive value of the three predefined summary measures. In line with the bidirectional observations made for the single items, the overall score was not associated with an improvement in outcome under chiropractic care. A slight tendency for improved outcome could be observed for increase in the chiropractor’s scores and the parental scores. All three interaction terms were negative, indicating an increasing advantage of chiropractic care with increasing score in the magnitude of a gain between 28 and 61 min in the reduction of crying time when comparing low and high score values. However, none of these interaction effects was statistically significant.

### Exploratory component

**Fig. 8:**
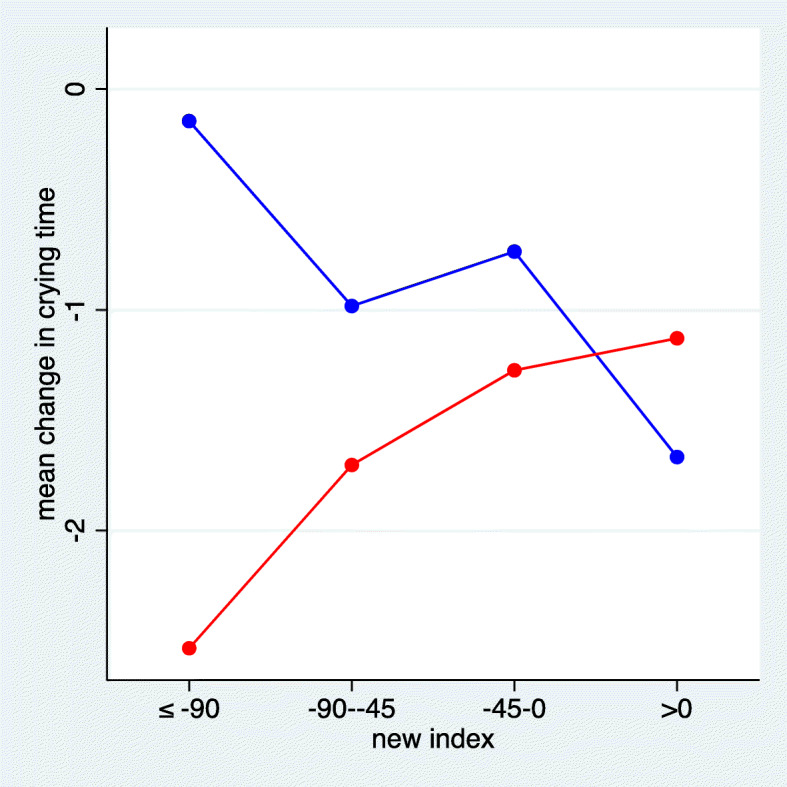
The mean change in crying time stratified by the new index based on all 13 musculoskeletal items. The values of the index refer to the predicted difference in crying time in minutes comparing active treatment with control (red = chiropractic care; blue = control group)

When combining all 13 musculoskeletal items into an index, the items were assigned the weights detailed in Additional Material Table 3. The weights indicate the additional decrease in crying hours following manual therapy when the item was present, i.e. a negative value indicates an advantage of the active treatment. When grouping the values of the index, we observed in Fig. 8 that children with a low value of the index had on average an additional gain of more than 2 h following manual therapy. However, the standard set up of the lasso selected no variables for a new index.

## Discussion

We were unable to approximate a latent variable reflecting the degree of benefit from manual therapy by a few preselected binary items assumed to be related to the musculoskeletal system. Although parents and chiropractors were able to answer the items without any indication of major conceptual problems, the items turned out to be quite independent of each other. Moreover, the observed associations with the gain from manual therapy were not pronounced and for some, even opposite in direction to what we expected. It is therefore neither surprising that the suggestions for predefined indices failed to show a strong association with the gain from manual therapy, nor that we failed in constructing a convincing new index. However, even if the preselected scores failed to show strong and statistically significant interactions, they all showed a negative interaction, i.e. children with many items tended to have a larger gain. The musculoskeletal items were all reported by the chiropractor based on visual inspection of the infants and interviews with their parents. The reliability of these items is unknown and more objective measurements might have provided a different answer.

We also considered six alternative baseline factors, apparently unrelated to the musculoskeletal system, as potential predictive factors, and the results indicated that the age of the child, the time since start of crying and the baseline level of crying were predictive factors. This is in line with the previous subgroup study, where the group with the smallest effect of treatment also included the oldest children [22].

To our knowledge, only one previous study has attempted to subgroup excessively crying infants into groups which might differ in response to chiropractic care [11] but that study was observational, and the groups were vaguely defined and thus difficult to replicate. They did however demonstrate significantly different treatment effects. The current study is the first attempt at a systematic identification of effect modifiers of chiropractic treatment for excessive crying due to infantile colic using an RCT design. The analyses were premeditated in the design of the RCT, but power calculation for the RCT was aimed at the overall effect, and thus potentially underpowered for these effect modification analyses.

A Cochrane review from 2012 identified three RCTs of chiropractic care on infantile colic and a later update provided no new knowledge [7, 8]. One of these RCTs found no evidence of effect [23], whereas the other two were suggestive of a beneficial effect [22, 24]. We also showed a beneficial effect in the current RCT, but it was considerably smaller than the other two trials [18]. The two RCTs which reported a larger effect of chiropractic treatment than our RCT (between group differences of 1.4 and 1.7 h/day compared to 0.5 h/day in our study), included children with a mean age of 4.9 and 5.9 weeks, respectively, whereas the mean age was 6.7 weeks in our study. This could support the theory that younger age/shorter duration of symptoms enhances the treatment effect. There were no differences in baseline crying levels among the studies, so a possible effect hereof could not be evaluated.

There are no indications in the results of the current study which can shed light on the reasons why the RCT by Olafsdottir et al. [23] provided a fundamentally different result than the other RCTs.

Our results confirm a diverse response to chiropractic care for the group of excessively crying infants and we still see some potential in identifying children for whom manual therapy could be particularly successful, but further research is needed to develop a more valid instrument for this. Combining musculoskeletal indicators did not provide the answer, indicating either that the dominant theory of chiropractic care only influencing the amount of crying in children with visible musculoskeletal problems is not valid; or that the identification of musculoskeletal indicators in this study failed. These were identified by a broad panel of both experienced chiropractors and maternal and child health nurses, who therefore were expected to represent a broad understanding of musculoskeletal problems in infants, but the reliability is uncertain. Importantly, asymmetrical head position/neck movement was also pointed out as an important factor by the chiropractor panel, but unfortunately this was not included in the data collection. It is possible that inclusion of this could have improved the prediction. Furthermore, it should be noted that the treating chiropractors were asked about their expectations prior to examining the child. Hence, we do not know whether the chiropractors’ expectations after examining the child were more valuable.

On the other hand, more general factors not related to the musculoskeletal system appeared more promising. The results suggest that manual therapy is particularly valuable if applied early in the course of symptoms and for children with a high level of baseline crying.

In future studies, the reliability of the musculoskeletal indicators should be investigated. Furthermore, the findings from this study should be followed up by investigations into the prognostic value of the identified effect modifiers, including those not reaching statistical significance, in larger cohorts in chiropractic practice of infants with excessive crying, and if possible, develop clinical prediction rules. It was clear, that the group of excessively crying infants was heterogenous and showed large variation in the response to chiropractic treatment. Therefore, to optimise care, it is important to identify both positive and negative indicators for a possible treatment effect.

## Conclusion

None of the preselected items or predefined indices were valuable for identification of infants with potentially larger gain from manual therapy than others. However, more hours of crying at baseline, short duration of symptoms and young age were all associated with an increased effect of chiropractic care; hours of crying at a statistically significant level. This may be a clinically relevant finding, suggesting that the most severely affected infants have the greatest potential for an advantage from manual therapy. This finding requires validation by further studies.

## Supplementary Information

**Additional file 1: Table 1.** Associations between the musculoskeletal items (Odds Ratios). **Table 2.** Association between the six additional baseline variables and with the musculoskeletal items (Pearson’s correlations). **Table 3.** Supplementary Table Weights. The weights assigned to each item in the new index together with their standard errors. The weights indicate the decrease (or increase) in the gain in reducing the crying time by manual therapy when the item is present.

## Data Availability

The datasets generated and/or analysed during the current study are not publicly available due to the policies of the Danish Data Protection Agency but are available from the corresponding author on reasonable request.

## Abbreviations

RCT: Randomised Controlled Trial
CI: Confidence interval
