# Supplementary material for: Identifying potential treatment effect modifiers of the effectiveness of chiropractic care to infants with colic through prespecified secondary analyses of a randomised controlled trial
Source: Chiropr Man Therap. 2021 Apr 19;29:16. doi: 10.1186/s12998-021-00373-6 (PMC8054382; doi:10.1186/s12998-021-00373-6)
Supplement: Supplementary file 1 — Additional file 1: Table 1. Associations between the musculoskeletal items (Odds Ratios). Table 2. Association between the six additional baseline variables and with the musculoskeletal items (Pearson’s correlations). Table 3. Supplementary Table Weights. The weights assigned to each item in the new index together with their standard errors. The weights indicate the decrease (or increase) in the gain in reducing the crying time by manual therapy when the item is present. [file 12998_2021_373_MOESM1_ESM.docx]

**Additional Material Table 1.** Associations between the musculoskeletal items (Odds Ratios)

|  | **resist prone** | **resist dressing** | **sudden crying** | **disturbed sleep** | **tense meals** | **fav. side meals** | **fav. side sleep** | **back extensions** | **asym. glut** | **asym. hips** | **asym. knees** | **c-curve** | **asym. back tonus** | **expectation** |
| --- | --- | --- | --- | --- | --- | --- | --- | --- | --- | --- | --- | --- | --- | --- |
| **resist prone** |  | 1,93 | 0,64 | 1,03 | 1,37 | 1,44 | 1,25 | 0,91 | 1,30 | 0,95 | 0,70 | 1,32 | 2,05 | 4,82 |
| **resist dressing** | 1,93 |  | 0,93 | 1,12 | 1,78 | 1,75 | 2,88 | 0,93 | 1,04 | 0,81 | 0,62 | 1,71 | 1,12 | 2,59 |
| **sudden crying** | 0,64 | 0,93 |  | 0,91 | 1,31 | 1,02 | 1,55 | 0,89 | 1,25 | 1,04 | 0,38 | 2,12 | 1,28 | 2,94 |
| **disturbed sleep** | 1,03 | 1,12 | 0,91 |  | 1,18 | 0,87 | 1,33 | 0,91 | 1,59 | 1,31 | 0,93 | 1,29 | 1,32 | 2,03 |
| **tense meals** | 1,37 | 1,78 | 1,31 | 1,18 |  | 1,74 | 1,25 | 1,54 | 0,97 | 0,85 | 0,52 | 1,33 | 1,33 | 2,88 |
| **fav. side meals** | 1,44 | 1,75 | 1,02 | 0,87 | 1,74 |  | 7,50 | 1,15 | 0,79 | 0,89 | 0,63 | 1,31 | 1,61 | 4,40 |
| **fav. side sleep** | 1,25 | 2,88 | 1,55 | 1,33 | 1,25 | 7,50 |  | 2,33 | 1,02 | 1,11 | 0,76 | 1,87 | 1,55 | 6,51 |
| **back extensions** | 0,91 | 0,93 | 0,89 | 0,91 | 1,54 | 1,15 | 2,33 |  | 0,98 | 1,12 | 0,53 | 1,51 | 2,55 | 4,94 |
| **asym. glut** | 1,30 | 1,04 | 1,25 | 1,59 | 0,97 | 0,79 | 1,02 | 0,98 |  | 7,84 | 2,01 | 0,76 | 1,74 | 1,10 |
| **asym. hips** | 0,95 | 0,81 | 1,04 | 1,31 | 0,85 | 0,89 | 1,11 | 1,12 | 7,84 |  | 36,11 | 1,60 | 1,48 |  |
| **asym. knees** | 0,70 | 0,62 | 0,38 | 0,93 | 0,52 | 0,63 | 0,76 | 0,53 | 2,01 | 36,11 |  | 0,89 | 0,55 | 2,20 |
| **c-curve** | 1,32 | 1,71 | 2,12 | 1,29 | 1,33 | 1,31 | 1,87 | 1,51 | 0,76 | 1,60 | 0,89 |  | 3,40 | 3,65 |
| **asym. back tonus** | 2,05 | 1,12 | 1,28 | 1,32 | 1,33 | 1,61 | 1,55 | 2,55 | 1,74 | 1,48 | 0,55 | 3,40 |  | 28,03 |
| **expectation** | 4,82 | 2,59 | 2,94 | 2,03 | 2,88 | 4,40 | 6,51 | 4,94 | 1,10 |  | 2,20 | 3,65 | 28,03 |  |

**Additional Material Table 2.** Association between the six additional baseline variables and with the musculoskeletal items (Pearson’s correlations)

|  | breast feeding | education mother | stress pregnancy or after | days since onset | child's age (days) | crying hours baseline |
| --- | --- | --- | --- | --- | --- | --- |
| breast feeding |  | 0,26 | -0,23 | -0,07 | -0,06 | 0,03 |
| education mother | 0,26 |  | -0,23 | 0,05 | 0,02 | -0,20 |
| stress pregnancy or after birt | -0,23 | -0,23 |  | 0,07 | 0,13 | 0,06 |
| days since onset | -0,07 | 0,05 | 0,07 |  | 0,84 | -0,07 |
| child's age (days) | -0,06 | 0,02 | 0,13 | 0,84 |  | -0,11 |
| crying hours baseline | 0,03 | -0,20 | 0,06 | -0,07 | -0,11 |  |
| resist prone lying | 0,03 | -0,05 | -0,11 | 0,04 | 0,02 | 0,08 |
| resist dressing | -0,13 | 0,05 | 0,08 | 0,02 | 0,01 | 0,14 |
| sudden crying | -0,02 | -0,05 | -0,13 | -0,01 | -0,07 | 0,02 |
| disturbed sleep | 0,01 | 0,02 | -0,04 | 0,05 | 0,00 | -0,24 |
| tense meals | 0,03 | 0,05 | 0,06 | 0,07 | 0,03 | 0,15 |
| fav. side meals | -0,08 | 0,06 | 0,00 | 0,04 | 0,06 | -0,04 |
| fav. side sleep | -0,12 | -0,01 | -0,06 | -0,06 | -0,09 | -0,02 |
| back extensions | -0,03 | -0,08 | -0,01 | 0,04 | 0,00 | 0,09 |
| asym. glut | 0,06 | 0,03 | 0,04 | 0,09 | 0,00 | 0,00 |
| asym. hips/knees | 0,04 | 0,02 | 0,07 | 0,07 | -0,01 | -0,06 |
| c-curve | -0,06 | -0,11 | -0,19 | -0,10 | -0,21 | 0,13 |
| asym. back tonus | 0,11 | -0,13 | -0,01 | -0,08 | -0,10 | 0,12 |
| expectation | 0,02 | 0,08 | -0,08 | 0,12 | 0,09 | 0,05 |

**Additional Material Table 3.** Supplementary Table Weights. The weights assigned to each item in the new index together with their standard errors. The weights indicate the decrease (or increase) in the gain in reducing the crying time by manual therapy when the item is present.

| **Item** | **weight SE** |
| --- | --- |
| Resist prone lying | -.3345888 .5276703 |
| Resist dressing | -1.020387 .5388339 |
| Sudden crying | -.7073081 .7490263 |
| Disturbed sleep | .7429450 .5473637 |
| Tense at meals | .9860229 .5245868 |
| Favourite side, meals | -.5848310 .5760523 |
| Favourite side, sleep | .5968338 .6020730 |
| Back extension | .2833471 .5649899 |
| Asym. gluteal muscles | .3292844 .5975482 |
| Asym. hips/knees | .5626108 .7279539 |
| C-curve | -1.160218 .6319039 |
| Asym. back tonus | -.035486 .5517493 |
|  |  |
